# Accuracy and Clinical Performance of Intraoral Scanners Compared to Conventional and Extraoral Impressions: An Umbrella Review

**DOI:** 10.7759/cureus.93202

**Published:** 2025-09-25

**Authors:** Rajeev Singh, Gaurang Mistry, Ashwini Kini, Rasha Ansari, Vibha Kailaje, Shresth Kapoor

**Affiliations:** 1 Department of Prosthodontics, DY Patil University School of Dentistry, Navi Mumbai, IND

**Keywords:** accuracy, digital impressions, extraoral scanners, intraoral scanners, prosthodontics, systematic review, umbrella review

## Abstract

Digital impression technologies have transformed modern dental workflows, with intraoral scanners (IOS) emerging as a prominent alternative to conventional elastomeric impressions and extraoral scanning systems. This umbrella review aims to comprehensively synthesize evidence from systematic reviews, meta-analyses, and umbrella reviews evaluating the accuracy, efficiency, and clinical applicability of IOS across various prosthodontic, implant, and orthodontic applications. A total of 10 reviews published between 2020 and 2024 were included, encompassing over 30 IOS models and a wide spectrum of clinical settings. Trueness and precision were the most frequently evaluated outcomes, with TRIOS 3 (3Shape, Copenhagen, Denmark) and Primescan (Dentsply Sirona, Charlotte, NC, USA) consistently ranking highest in complete-arch accuracy. Compared to traditional impressions, IOS generally reduced procedural time and enhanced patient comfort, although accuracy in partially edentulous and edentulous arches remained a limiting factor. Meta-analytical findings supported the superior performance of certain IOS platforms over others and affirmed the time-saving benefits of digital scanning. However, most reviews relied heavily on in-vitro studies, and few conducted robust risk of bias assessments or used the Grading of Recommendations Assessment, Development, and Evaluation (GRADE) approach to assessing certainty of evidence. Assessment of Multiple Systematic Reviews-2 (AMSTAR-2) analysis revealed only two reviews met high-quality methodological standards, while others showed moderate to critically low ratings. The findings underscore the clinical advantages of IOS while highlighting the need for standardized protocols and higher-quality evidence in complex restorative scenarios.

## Introduction and background

The incorporation of digital technologies into dentistry has revolutionized traditional clinical workflows, particularly in the realm of dental impressions [1]. Among these innovations, digital impression systems have emerged as powerful tools offering enhanced accuracy, reduced chairside time, greater patient comfort, and seamless integration with computer-aided design/computer-aided manufacturing (CAD/CAM) workflows [1,2]. The origins of digital impression techniques date back to the 1980s with the development of the earliest intraoral scanners (IOS) [2,3]. Since then, progressive advancements in hardware components, imaging software, and data processing algorithms have expanded the clinical utility of IOS across multiple disciplines, including prosthodontics, orthodontics, restorative dentistry, and implantology [1-3].

Digital impression techniques can be broadly categorized into IOS and extraoral scanners (EOS) [2,4,5]. Intraoral scanners are handheld optical devices that acquire three-dimensional (3D) images of dental structures and surrounding tissues directly from within the oral cavity [6]. These scanners generate virtual models in real time, eliminating the need for conventional trays, impression materials like alginate or polyvinyl siloxane, and associated steps such as disinfection and stone pouring [6,7]. In contrast, extraoral scanners are benchtop or laboratory-based systems that digitize physical models or traditional impressions after they have been removed from the patient’s mouth [8]. Although EOS can achieve high levels of precision under standardized laboratory conditions, they are inherently dependent on the quality and dimensional stability of the physical impressions, making it susceptible to errors from material distortion or voids introduced during impression-taking or casting [9].

The accuracy of any dental impression, whether digital or conventional, is typically defined by two primary components: trueness, or how closely the scan replicates the true geometry of the target object, and precision, or the consistency of scan results under repeated measurements [10]. These parameters are critical for achieving optimal fit, occlusion, and functional stability in prosthetic and implant-supported restorations. Errors at this foundational stage can propagate through subsequent fabrication steps, leading to ill-fitting restorations, occlusal interferences, increased chairside adjustments, and reduced patient satisfaction [10,11].

Several factors influence the accuracy of both intraoral and extraoral scanning systems [7]. These include optical resolution, software-based image alignment algorithms, scanning strategy, operator proficiency, intraoral conditions such as saliva or bleeding, patient movement, lighting variability, and limited access to posterior regions [12]. IOS, despite their continuous technological refinement, remain sensitive to clinical handling and intraoral environmental variables [12]. Conversely, EOS devices are less affected by oral conditions but remain dependent on the quality of the traditional impressions they digitize. Moreover, the accuracy of digital scanners is highly context-dependent and may vary across clinical scenarios, including single-unit crowns, full-arch prostheses, implant workflows, and orthodontic applications [13].

Given the growing adoption of IOS in everyday dental practice and the rapidly evolving scanner technologies, it is imperative to periodically re-evaluate their accuracy and performance compared to conventional and extraoral alternatives [10-13]. While numerous studies and reviews have explored these comparisons, the evidence remains fragmented due to variations in clinical protocols, scanner models, outcome definitions, and methodological rigor. Additionally, the frequent introduction of new scanner generations and software updates necessitates ongoing evidence consolidation.

Accordingly, the present umbrella review was undertaken to systematically identify, evaluate, and synthesize the findings from existing systematic reviews, meta-analyses, and umbrella reviews on intraoral scanning accuracy and clinical performance. The objective is to provide clinicians and researchers with a consolidated and up-to-date evidence base to guide informed decision-making regarding the use of IOS in modern restorative and prosthodontic dentistry.

## Review

Methodology

Study Design, Protocol, and Registration

This umbrella review was conducted to systematically evaluate and synthesize existing high-level evidence regarding the accuracy, clinical reliability, and practical applicability of IOS compared to conventional and extraoral impression techniques in dental practice. The review adhered to the Preferred Reporting Items for Systematic Reviews and Meta-Analyses (PRISMA) 2020 guidelines [14]. To ensure transparency and reproducibility, the review protocol was prospectively registered in the International Prospective Register of Systematic Reviews (PROSPERO) under the registration number CRD42024573970.

Eligibility Criteria

Only systematic reviews (SRs), systematic reviews with meta-analyses (SRMAs), and umbrella reviews published between January 2015 and March 2024 were included. Eligible reviews were required to evaluate the performance of intraoral scanners either as standalone technologies or in direct comparison with conventional elastomeric impression methods or extraoral digital scanning techniques. Included reviews had to report on one or more outcomes of interest, such as accuracy (trueness and precision), reproducibility, scanning time, patient comfort, prosthetic fit, or clinical workflow efficiency. All included reviews had to be peer-reviewed, written in English, and provide a transparent description of methodology, including eligibility criteria, database sources, and risk of bias assessment methods.

Reviews were excluded if they were narrative in nature, lacked systematic methodology, or focused solely on primary studies without synthesizing existing review-level evidence. Expert opinions, case series, editorials, and conference abstracts without full-text availability were also excluded, as were non-English language publications.

Research Question and Conceptual Framework

The research question was framed using the PICOS model (Population, Intervention, Comparison, Outcomes, and Study design), which guided the formulation of inclusion criteria and data extraction. The population of interest included patients undergoing dental impressions for a variety of clinical applications, such as single crowns, multiple-unit fixed restorations, full-arch implant-supported prostheses, removable complete dentures, and orthodontic study models or aligners. The intervention comprised intraoral scanners or digital impression techniques, while comparators included conventional impression methods using materials such as polyether or polyvinyl siloxane, or extraoral scanning of physical models using benchtop scanners. Outcomes of interest included trueness and precision (accuracy), procedural time, patient preference, and prosthetic success. Only SRs, SRMAs, and umbrella reviews were considered eligible for inclusion under the study design domain.

Literature Search Strategy

A comprehensive electronic literature search was conducted across six major databases: PubMed/MEDLINE, Cochrane Library, Scopus, Embase, Web of Science, and Google Scholar. The search covered the period from January 2015 through March 2024. Search terms included combinations of Medical Subject Headings (MeSH) and free-text keywords such as “intraoral scanner,” “digital impressions,” “accuracy,” “trueness,” “precision,” “implant scanning,” “conventional impressions,” and “prosthodontics digital workflow.” Boolean operators (“AND,” “OR”) were applied to combine relevant concepts and optimize retrieval. Filters were applied to limit results to English-language articles and review-type publications. Additional manual searches of the reference lists of included reviews were performed to identify any missed eligible publications.

Duplicate records were identified and removed using reference management software (Zotero, Corporation for Digital Scholarship, Fairfax, VA, USA). Two independent reviewers conducted the screening process in three stages: title screening, abstract screening, and full-text evaluation. Any disagreements were resolved by discussion or by consulting a third senior reviewer when necessary.

Data Extraction and Management

For each included review, data were extracted using a standardized and pre-piloted data extraction form. Extracted variables included first author and year of publication, review type (systematic review, meta-analysis, or umbrella review), protocol registration status, databases searched, last date of search and coverage period, clinical applications addressed, intraoral scanner models evaluated, comparator techniques, study designs of included primary studies, number of primary studies, total participant/sample size (if reported), key outcome measures (e.g., trueness, precision, scanning time), and main conclusions. Where applicable, pooled effect estimates, confidence intervals, and heterogeneity statistics (e.g., I², τ²) were also extracted.

Risk of Bias Assessment

The methodological quality of all included systematic reviews was assessed using the Assessment of Multiple Systematic Reviews-2 (AMSTAR-2) instrument [15]. This tool evaluates the quality of systematic reviews across 16 domains, including protocol registration, comprehensiveness of the literature search, risk of bias assessment in included studies, consideration of publication bias, justification of study design selection, duplicate screening and extraction, and appropriateness of synthesis methods. Each review was rated domain-by-domain and assigned an overall confidence rating as high, moderate, low, or critically low. The assessment was independently conducted by two reviewers with expertise in evidence synthesis. Any discrepancies were resolved through consensus discussion, with arbitration by a third reviewer when needed. The results of the domain-wise assessment were summarized in tabular and heatmap formats to aid interpretation.

Data Synthesis

The extracted data were synthesized qualitatively, with emphasis on patterns and trends across the included reviews regarding scanner accuracy, clinical applicability, time efficiency, patient-reported outcomes, and prosthetic fit. Quantitative meta-analysis was not performed at the umbrella review level due to heterogeneity in outcome measures, scanner models, clinical settings, and statistical methods used across the reviews. Instead, reported effect sizes and meta-analytic outcomes from the original SRMAs were summarized narratively and integrated into the overall synthesis. Methodological heterogeneity, including differences in study design, scanner generations, clinical protocols, and evaluation criteria, was examined and described qualitatively. Publication bias assessment was captured from the included reviews where reported, although none conducted formal tests such as Egger’s regression or funnel plot asymmetry analysis.

Results

Overview of Included Reviews

**Figure 1:**
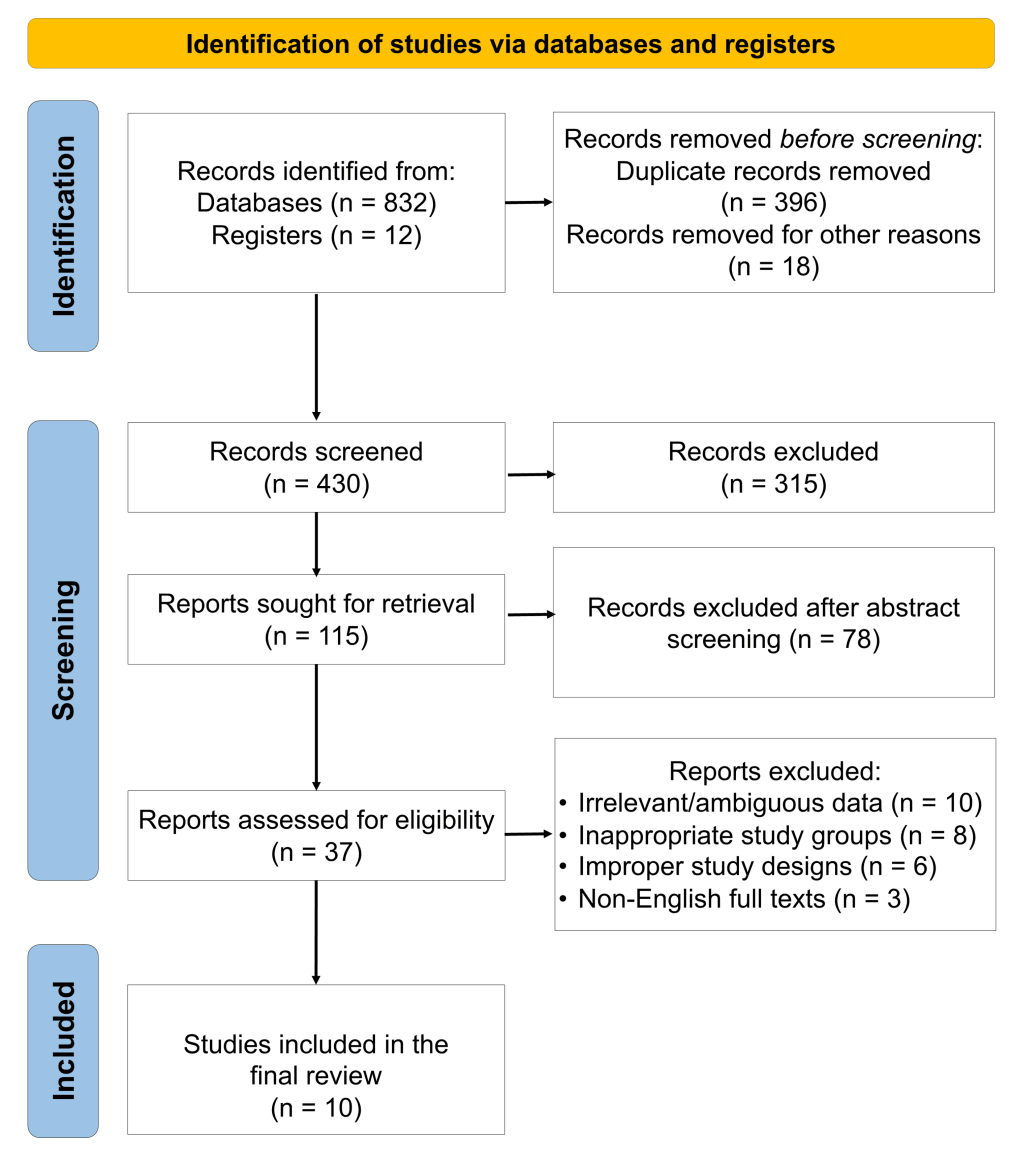
PRISMA flow diagram indicating the selection process of the articles in the present systematic review PRISMA: Preferred Reporting Items for Systematic Reviews and Meta-Analyses

A total of 10 systematic reviews were included in this umbrella review, published between 2020 and 2024; the process is depicted in Figure 1.

These comprised seven conventional SRs, two systematic reviews with pairwise meta-analyses (Kachhara et al., 2020; Ma et al., 2023), and one systematic review with network meta-analysis (Vitai et al., 2023) [16-25]. Four of the reviews were registered in the PROSPERO database. The data extracted from the included systematic reviews are collectively displayed in Table 1.

**Table 1:** Data extracted from the included systematic reviews SR: systematic review; MA: meta-analysis; NMA: network meta-analysis; IOS: intraoral scanner; RCT: randomized controlled trial; CI: confidence interval; RoB: risk of bias; ROBINS‑I: Risk of Bias in Non‑randomized Studies of Interventions; NOS: Newcastle-Ottawa Scale; QUADAS-2: Quality Assessment of Diagnostic Accuracy Studies-2; MINORS: Methodological Index for Non-Randomized Studies; MD: mean difference; PROMs: patient-reported outcome measures; PVS: polyvinyl siloxane; NR: not reported; ISB: implant scan body; MAD: mean absolute deviation; RMS: root mean square

Author (year)	Review type	Protocol registration	Databases & sources searched	Last search date & coverage years	Clinical application(s)	IOS models/generations	Comparator technique(s)	Study designs included	Number of primary studies	Total participants/scans/units	Key outcome measures	Meta-analysis conducted?	Pooled effect estimate(s) ± 95% CI	Heterogeneity stats (I², χ² p, τ²)	RoB tool for primary studies	Overall RoB judgement	Conclusive findings
Kachhara et al. (2020) [16]	SR + MA	Not reported	PubMed; Google Scholar; Cochrane Library; Web of Science; EMBASE; Scopus	Search to Feb 2019; coverage 2014–2019	Full‑arch multi‑implant impressions	Lava COS; True Definition; CEREC Bluecam/Omnicam; iTero; ZFX Intrascan; 3D Progress; Trios/Trios 3	None (comparisons among IOS)	In vitro & in vivo incl. RCTs	8	NR (model‑based)	Accuracy (distance deviation), trueness, precision	Yes	Trios 3 superior to CEREC Omnicam (z = 3.53)	Precision I² low; trueness I² high (values NR)	None stated	High	Advanced IOS (Trios 3) most accurate; operator experience influences results
Siqueira et al. (2021) [19]	SR	PROSPERO CRD42020187021	PubMed/MEDLINE; Embase; Cochrane Central; ClinicalTrials.gov; OpenGrey; manual journal searches	Search to 25 Jul 2021; no start limit stated	Fixed prosthodontics & implant restorations (quadrant & full‑arch)	Trios; iTero; Lava COS; Omnicam; Bluecam; 3M True Definition; Carestream CS 3600	Conventional polyether/PVS impressions	RCTs; prospective clinical studies	17	437 patients (430 IOS; 370 CI)	Procedure working time; PROMs; prosthodontic fit	No	—	—	Cochrane RoB (RCTs); NOS (non‑RCTs)	Mixed (blinding high risk; non‑RCTs moderate)	IOS consistently faster and preferred by patients; provides reliable prosthodontic outcomes comparable to conventional
Afrashtehfar et al. (2022) [20]	Rapid umbrella SR	Not reported	PubMed/MEDLINE; Cochrane Database of Systematic Reviews; manual reference screening	Search in Mar 2021; coverage 2020–2021	Fixed & removable prosthodontics, single‑unit to full‑arch, implant & edentulous scenarios	Multiple IOS systems (e.g., Trios, iTero, active wavefront sampling IOS)	Conventional impressions	Systematic reviews (clinical & in vitro primary studies)	11 secondary studies	NR	Trueness, precision, accuracy, patient preference, time efficiency	No	—	—	Not reported	—	Lab data: IOS ≈ conventional; clinical up to 3‑unit similar; conventional preferred for extensive cases; IOS faster & preferred by patients; better reporting & more RCTs needed
Alassiry et al. (2023) [21]	SR	Not reported	PubMed; Scopus; Google Scholar; Embase; Web of Science; Cochrane Central	Search to 31 Dec 2022; inception–2022	Orthodontic study models & aligner fabrication	Multiple IOS systems (Trios 3, CS 3600, iTero, CEREC, Lythos)	Conventional elastomeric/alginate impressions & cast digitization	Clinical in vivo & ex vivo comparative studies	35	NR	Accuracy; reproducibility; scanning time; patient comfort; operator experience	No	—	—	Not reported	—	IOS time‑efficient with greater patient comfort; accuracy deemed clinically acceptable for orthodontic applications though evidence mixed; operator learning curve noted
Ma et al. (2023) [17]	SR + MA	Not reported	PubMed; EMBASE; Cochrane Library; manual reference & conference searches	Search to Aug 2023; coverage 1989–2023	Single, partial & full‑arch implant restorations	Multiple IOS (Trios, iTero, True Definition, Lava COS, CS 3600)	Conventional elastomeric impressions	RCTs; comparative clinical; single‑arm trials	10	NR	Accuracy (trueness, precision); procedure time; marginal bone loss; prosthetic survival	Yes	Procedure time MD 8.59 min (6.78–10.40); bone loss 12 mo MD 0.03 mm (‑0.08–0.14)	NR	Cochrane RoB; MINORS	Mixed	IOS clinically accurate and time‑efficient; accuracy depends on scan strategy; trueness/precision for partial/full arches still uncertain; evidence limited
Pullishery et al. (2023) [22]	SR	Not reported	PubMed; Scopus; EMBASE; Cochrane Oral Health Group; Dentistry and Oral Science Source; Google Scholar; manual searches	Search to Apr 2023; coverage 2014–2023	Implant‑supported fixed partial dentures	Trios 3; iTero	Conventional implant impression (polyvinyl siloxane/polyether)	2 RCTs; 6 prospective clinical studies	8	NR	Accuracy (trueness, precision); fabrication time; patient preference	No	—	—	Cochrane RoB; NOS	Mixed	IOS preferred by patients, time‑efficient; accuracy comparable; additional in vivo evidence required
Shah et al. (2023) [23]	SR	PROSPERO CRD42020188765	PubMed; EBSCOhost; Cochrane Library; Google Scholar	Search to 1 Dec 2022; coverage 2010–2022	Digital impressions (partial vs full arch)	Multiple IOS scanners (various)	Extraoral scanners	In vivo & in vitro comparative studies	10	NR	Accuracy (trueness, precision)	No	—	—	Modified MINORS	Low	IOS more accurate for partial arch; both scanner types clinically acceptable; more in vivo full‑arch studies needed
Srivastava et al. (2023) [24]	SR	PROSPERO CRD42021289821	PubMed; Scopus; Web of Science	Search to Aug 2023; no date limit	Completely edentulous arches (complete dentures)	Multiple IOS (Trios 3, Lava COS, iTero, CS3500, CEREC Omnicam, etc.)	Conventional impressions (ZOE, PVS, elastomers)	Clinical non‑randomized studies & in vitro	12	126 patients; 50 models	Accuracy (trueness, precision)	No	—	—	QUADAS‑2	Low	IOS scans clinically acceptable for denture‑bearing areas; discrepancies greater in mobile tissues and borders; careful interpretation needed
Vitai et al. (2023) [18]	SR + NMA	PROSPERO CRD42021281989	Medline; Scopus; Web of Science; EMBASE; Cochrane Central	Search to 23 Oct 2021; inception–2021	Complete‑arch dentate/edentulous ± implants	26 IOS (Trios 3, Primescan, Omnicam, iTero, Lava COS, etc.)	Reference lab/industrial scanner STL	In vitro & limited clinical diagnostic accuracy studies	114 (53 in NMA)	NR (mostly model‑based)	Trueness; precision (MAD & RMS)	Yes (network MA)	NMA rank: Primescan & Trios 3 highest accuracy; pooled comparisons vary	Consistency tests acceptable; I² NR	QUADAS‑2	Unclear–high	IOS accuracy arch‑dependent; several IOS (Primescan, Trios 3) clinically acceptable for dentate arches; none acceptable for partially edentulous arches; selection should consider arch type
Gehrke et al. (2024) [25]	SR	PROSPERO 451137	MedLine; PubMed; Scopus; manual reference screening	Search to Mar 2023; coverage 2015–2023	Implant dentistry – scan body accuracy (single & short‑span)	Multiple IOS (Primescan, iTero, Medit i500, Vatech EZ scan, Trios 3, etc.)	Conventional implant impressions & inter‑IOS comparisons	In vitro (13) & clinical investigations (3)	16	79 patients (clinical); remainder in vitro	Trueness; precision; linear & angular deviations	No	—	—	ROBINS‑I	Low	ISB position, design, material, and IOS/scanning strategy affect accuracy; IOS with ISBs comparable to conventional in single/short spans; insufficient in vivo data for extensive arches

Geographic and Temporal Distribution

While exact country-specific distributions were not provided by all reviews, the primary studies synthesized originated from various parts of the world. Italy contributed the largest number of studies (n=24), followed by Turkey (n=21), the United States (n=16), Germany (n=14), South Korea (n=12), India (n=11), and Brazil (n=8). Several additional studies originated from countries such as Spain, the United Kingdom, Japan, Iran, and China, underscoring the global relevance of intraoral scanning in dental practice [16-25].

Clinical Applications and Scope

The included reviews addressed a wide range of clinical applications for IOS. These included implant-supported prosthodontics (n=6), fixed prosthodontics involving single-unit, quadrant, or full-arch restorations (n=3), removable complete denture fabrication (n=1), and orthodontic aligner production or digital model analysis (n=1). Some reviews focused on specific clinical workflows, such as the accuracy of scan body positioning in implants (Gehrke et al., 2024), while others provided a broader comparison across digital impression technologies [25].

Types of Intraoral Scanners and Comparators

Across the reviews, over 30 different intraoral scanner models were evaluated. The most frequently reported devices were 3Shape TRIOS (especially TRIOS 3; 3Shape, Copenhagen, Denmark), iTero (Align Technology, San Jose, CA, USA), Primescan (Dentsply Sirona, Charlotte, NC, USA), Carestream CS 3600 (Carestream Dental, Atlanta, GA, USA), CEREC Omnicam (Dentsply Sirona, Charlotte, NC, USA), and Lava COS/3M True Definition (3M ESPE, St. Paul, MN, USA). Comparator techniques included conventional elastomeric impressions using polyether or polyvinyl siloxane (PVS) (n=7), extraoral/laboratory scanners (n=2), and inter-IOS comparisons only (n=1). Most studies focused on IOS versus conventional workflows, although some reviews, such as Kachhara et al. (2020), exclusively compared multiple IOS platforms [16].

Outcome Measures

All ten reviews reported on accuracy outcomes, primarily trueness and precision, often measured in micrometres using root mean square (RMS) deviation or mean absolute deviation (MAD). Additional outcomes included procedure working time (n=3), patient-reported comfort and preference (n=4), prosthodontic fit (n=2), prosthetic survival (n=1), marginal bone loss (n=1), and operator experience (n=2). Patient satisfaction was consistently higher with IOS in all reviews that addressed this domain, and chairside time was generally reduced compared to conventional impression methods.

Meta-Analytical Findings

Three reviews conducted quantitative syntheses. Kachhara et al. (2020) found that the TRIOS 3 scanner demonstrated significantly higher accuracy than CEREC Omnicam (z = 3.53) [16]. Ma et al. (2023) reported that intraoral scanning reduced clinical procedure time by an average of 8.59 minutes (95% CI: 6.78 to 10.40), while no significant difference in marginal bone loss was noted at 12 months between IOS and conventional methods [17]. Vitai et al. (2023) conducted a network meta-analysis comparing 26 scanners, ranking Primescan and TRIOS 3 as the most accurate for complete-arch scans in dentate arches; however, none of the IOS were found sufficiently accurate for partially edentulous arches [18].

Study Designs in Primary Evidence

Across all reviews, the primary studies included a mix of in vitro experimental designs, prospective clinical trials, and randomized controlled trials. While all reviews included in vitro studies, only three (Siqueira et al., 2021; Ma et al., 2023; Pullishery et al., 2023) included randomized controlled trials, and only two of those performed a formal risk of bias analysis using the Cochrane tool [17,19,22]. Most of the clinical data came from non-randomized prospective studies and model-based assessments.

Risk of Bias and Certainty of Evidence

Risk-of-bias assessments were reported inconsistently. Three reviews used validated tools such as Quality Assessment of Diagnostic Accuracy Studies-2 (QUADAS-2), Risk of Bias in Non-randomized Studies of Interventions (ROBINS-I), or Methodological Index for Non-Randomized Studies (MINORS) to assess primary studies, while others provided narrative quality commentary [26-28]. Only a few reviews (e.g., Gehrke et al., 2024; Srivastava et al., 2023) judged the risk of bias as low [24,25]. None of the reviews used the Grading of Recommendations Assessment, Development, and Evaluation (GRADE) approach to assessing certainty of evidence. Publication bias assessments were absent from all included reviews, and AMSTAR-2 ratings were generally not reported [15].

AMSTAR Grading of Evidence

The methodological quality of the included systematic reviews was evaluated using the AMSTAR-2 tool (Table 2), revealing considerable variability across studies [15]. Two of the included reviews, performed by Kachhara et al. (2020) and Alassiry et al. (2023), were rated as high quality, meeting nearly all critical and non-critical AMSTAR domains, including clearly defined research questions, comprehensive literature searches, duplicate screening and extraction, risk of bias assessments, and appropriate synthesis methods [16,21]. Six reviews were rated as moderate in quality, typically due to limitations such as the absence of prior protocol registration, incomplete reporting of funding sources for primary studies, or failure to formally assess publication bias. One review, Vitai et al. (2023), received a critically low rating due to the omission of risk of bias assessment in included studies and limited methodological transparency in the network meta-analysis [18]. Another review (Gehrke et al., 2024) showed methodological soundness in most domains but lacked a formal assessment of heterogeneity and funding, leading to a moderate rating [25]. Notably, only a minority of reviews reported on publication bias or employed tools such as GRADE for assessing certainty of evidence [29]. These findings underscore the need for improved methodological rigor and reporting standards in future systematic reviews evaluating intraoral scanners, particularly in domains critical to the credibility and applicability of evidence synthesis.

**Table 2:** Rating of evidence using AMSTAR-2 tool AMSTAR-2: Assessment of Multiple Systematic Reviews-2; RQ: research question; RoB: risk of bias; MA: meta-analysis; NMA: network meta-analysis; NA: not applicable

Title (author, year)	RQ defined	Protocol registered	Study design justified	Comprehensive search	Selection in duplicate	Extraction in duplicate	List of studies provided	Characteristics described	RoB assessed in primaries	MA methods appropriate	Impact of RoB on results	RoB considered in discussion	Funding of primaries reported	Conflicts of interest declared	Publication bias assessed	Heterogeneity explained	Overall rating
Kachhara et al. (2020) [16]	Yes	No	Yes	Yes	Yes	Yes	Yes	Yes	Yes	Yes	Yes	Yes	No	Yes	Yes	Yes	High
Siqueira et al. (2021) [19]	Yes	Yes	Yes	Yes	Yes	Yes	Moderate	Yes	Yes	Moderate	Yes	Yes	No	Yes	No	Moderate	Moderate
Afrashtehfar et al. (2022) [20]	Yes	No	Yes	Moderate	Yes	Yes	Moderate	Yes	Yes	Moderate	Yes	Yes	No	Yes	No	Moderate	Moderate
Alassiry et al. (2023) [21]	Yes	No	Yes	Yes	Yes	Yes	Yes	Yes	Yes	Yes	Yes	Yes	Yes	Yes	Yes	Yes	High
Ma et al. (2023) [17]	Yes	No	Yes	Yes	Yes	Yes	Yes	Yes	Yes	Yes	Yes	Yes	No	Yes	No	Yes	Moderate
Pullishery et al. (2023) [22]	Yes	No	Yes	Yes	Yes	Yes	Yes	Yes	Yes	NA	Moderate	Yes	No	Yes	No	Moderate	Moderate
Shah et al. (2023) [23]	Yes	Yes	Yes	Yes	Yes	Yes	Yes	Yes	Yes	NA	Moderate	Yes	No	Yes	No	Moderate	Moderate
Srivastava et al. (2023) [24]	Yes	Yes	Yes	Yes	Yes	Yes	Yes	Yes	Yes	NA	Moderate	Yes	No	Yes	No	Moderate	Moderate
Vitai et al. (2023) [18]	Yes	Yes	Yes	Yes	Yes	Yes	Yes	Yes	No	Yes (NMA)	No	No	No	Yes	No	Yes	Critically low
Gehrke et al. (2024) [25]	Yes	Yes	Yes	Moderate	Yes	Yes	Yes	Yes	Yes	Moderate	Yes	Yes	No	Yes	No	Moderate	Moderate

Discussion

The present umbrella review consolidates and synthesizes high-level evidence from systematic reviews and meta-analyses assessing the accuracy, clinical efficiency, and patient-centered outcomes of IOS in comparison to conventional and EOS techniques. While digital impression technologies have increasingly permeated clinical practice over the past decade, this review attempts to clarify their relative advantages, limitations, and applicability across various prosthodontic, orthodontic, and implant-supported workflows [1-5].

The evidence across the included reviews consistently suggests that intraoral scanners offer high accuracy, particularly for single-unit and quadrant-level applications [16-25]. Among the evaluated devices, models such as TRIOS 3 and Primescan have emerged as the most reliable for complete-arch scans in dentate arches. However, the comparative performance in partially edentulous and fully edentulous arches remains less favorable, as highlighted by the network meta-analysis by Vitai et al. (2023), where IOS failed to demonstrate adequate accuracy in such complex anatomical scenarios [18]. However, these findings should be interpreted with caution because their review received a ‘critically low’ AMSTAR-2 rating due to the absence of a formal risk-of-bias assessment and limited methodological transparency. While the network meta-analysis provides useful preliminary insights, its conclusions regarding the limited accuracy of IOS in partially edentulous arches cannot be considered definitive without corroboration from higher-quality evidence. This discrepancy may be attributed to the challenges posed by soft tissue mobility, saliva contamination, limited visual markers, and difficulties in scanning body alignment in implant-supported prostheses, especially in edentulous arches [30,31].

Another critical insight pertains to time efficiency and patient experience. The majority of reviews reporting on procedural duration and patient comfort noted that IOS reduced chairside time and improved patient tolerance relative to traditional impression techniques [16-25]. These findings have significant implications in routine practice, where procedural efficiency, especially in geriatric or anxious patient populations, can directly influence treatment acceptability and adherence [32]. Nevertheless, a caveat remains regarding the initial learning curve and operator dependency associated with IOS, particularly in achieving optimal scanning strategies for full-arch or posterior regions.

It is noteworthy that despite the widespread inclusion of in vitro studies in most systematic reviews, clinical data remain somewhat limited, with relatively few high-quality randomized controlled trials available. Consequently, while in vitro findings support high trueness and precision of IOS, the translation of these findings into clinical predictability is nuanced, depending on patient-related, operator-related, and environmental variables. Moreover, only a minority of included reviews conducted formal risk-of-bias assessments or applied GRADE criteria to evaluate the certainty of evidence, potentially limiting the interpretive strength of some conclusions [29].

The observed heterogeneity across reviews can be attributed to several technical and procedural factors. Different scanner technologies, such as confocal microscopy, triangulation, and active wavefront sampling, use distinct optical principles that inherently affect trueness and precision. The heterogeneity in scanner generations, software updates, reference measurement methods, and scanning protocols across studies further complicates direct comparisons [16-25]. Variations in proprietary software algorithms for image stitching and data processing further influence the fidelity of the digital model. In addition, operator-related factors, including scanning strategies, angulation, and experience level, contribute to differences in outcome measures across studies. These device- and technique-specific variables complicate direct comparisons and preclude meaningful quantitative synthesis. Additionally, variability in outcome definitions makes it difficult to perform pooled analyses with consistent thresholds, especially in quantifying accuracy through different geometric deviation measures. The absence of standardization in accuracy reporting and evaluation protocols highlights an urgent need for uniformity in future investigations. 

The methodological assessment using AMSTAR-2 revealed variability in review quality [15]. Only two reviews achieved a high rating, while the rest demonstrated moderate to critically low confidence, primarily due to missing elements such as protocol registration, transparent risk of bias assessments, and publication bias analysis [16,17]. This inconsistency emphasizes the need for stricter adherence to established reporting standards in future systematic reviews and meta-analyses. It is noteworthy that although our search covered the period from 2015 to 2024, all ten included reviews were published between 2020 and 2024. This temporal clustering likely reflects a rapid maturation of intraoral scanner technology and a corresponding expansion of primary research in the past five years. The concentration of evidence synthesis in this short period also suggests a growing recognition of the need for rigorous, high-level reviews as IOS adoption accelerates in clinical practice. This recent surge underscores the dynamic nature of the field and highlights the importance of ongoing updates to capture emerging data.

Overall, IOS demonstrates significant clinical potential, particularly in improving patient comfort and procedural efficiency, while maintaining adequate accuracy for most restorative and orthodontic indications. However, their utility in complex prosthodontic or implant cases requires further validation through high-quality clinical trials, particularly those involving edentulous arches. Continued advancements in hardware, software, and operator training, coupled with better standardized evidence synthesis methods, will be essential in shaping the future of digital impression technologies in dentistry. Despite the abundance of data on accuracy, procedural time, and patient preference, the systematic reviews provide little synthesized evidence on more definitive clinical or economic outcomes. Key gaps include long-term survival and success rates of prostheses fabricated from digital versus conventional impressions, cost-effectiveness analyses comparing these workflows, and the practical implications of scanner-milling unit interoperability (open versus closed systems). Future reviews should explicitly incorporate these endpoints to better guide real-world decision-making and economic policy in digital dentistry.

Limitations of the Present Umbrella Review

This umbrella review has certain limitations that should be acknowledged. The synthesis relied entirely on previously published systematic reviews and meta-analyses; therefore, its conclusions are constrained by the methodological quality, scope, and reporting standards of the included reviews. Furthermore, because the existing reviews focus predominantly on accuracy, procedural time, and patient preference, this umbrella review inherits a narrow evidentiary scope. High-level data on long-term prosthesis survival, cost-effectiveness, and cross-platform interoperability remain sparse, limiting the ability to draw conclusions on these clinically critical outcomes. Although AMSTAR-2 was applied, most reviews were rated as moderate to critically low in quality, which reduces confidence in the pooled evidence. The heterogeneity in scanner generations, clinical settings, comparator techniques, and outcome definitions across reviews precluded quantitative synthesis at the umbrella level and limited direct comparability. Several included reviews combined in vitro and in vivo data without clear stratification, potentially inflating precision estimates. Additionally, none of the included reviews conducted formal publication bias analyses or certainty of evidence grading (e.g., GRADE), which restricts the ability to judge the robustness and applicability of the findings. Furthermore, as only English-language reviews were considered, there is a possibility of language bias and omission of relevant evidence. These limitations highlight the need for more standardized, high-quality systematic reviews and meta-analyses, with transparent risk-of-bias assessments, consistent outcome definitions, and formal certainty grading, to strengthen the evidence base for intraoral scanners.

The present umbrella review also exposed several critical gaps that should guide future primary research and evidence syntheses. First, there is a clear need for long-term (≥ 5-year) randomized controlled trials comparing the survival and success of restorations fabricated using IOS versus conventional impressions. High-quality in vivo studies evaluating IOS accuracy and predictability in fully edentulous and complex multi-implant cases remain scarce. While the time efficiency of IOS is well supported, comprehensive economic analyses are necessary to determine true cost-effectiveness for both clinicians and institutions. Additionally, the absence of standardized protocols for measuring and reporting IOS accuracy hampers cross-study comparison and meta-analysis. Developing universally accepted definitions and metrics for trueness and precision should therefore be a priority for the field

## Conclusions

The present umbrella review consolidates current high-level evidence comparing intraoral scanners with conventional and extraoral impression techniques, highlighting the consistent advantages of IOS in reducing chairside time, improving patient comfort, and achieving clinically acceptable accuracy in most restorative and prosthodontic applications. While certain IOS models, particularly newer-generation devices, demonstrate superior trueness and precision in dentate and short-span scenarios, limitations remain in complete-arch, edentulous, and implant-supported prostheses where conventional or hybrid workflows may still be preferable. Variability in study methodologies, operator experience, and outcome assessment underscores the need for standardized protocols and robust clinical trials to strengthen the evidence base. Ultimately, IOS technology offers a reliable and patient-friendly alternative to traditional impressions, but its optimal integration into clinical practice should be guided by case-specific requirements, practitioner expertise, and ongoing technological advancements.
